# POCUS in Differentiating Etiology of Acute Scrotum

**DOI:** 10.24908/pocusj.v11i01.19770

**Published:** 2026-04-22

**Authors:** Matthew Starr, Jamie Baydoun

**Affiliations:** 1 Department of Emergency Medicine, University of Nevada, Las Vegas, NV, USA; 2 Emergency Department, University Medical Center of Southern Nevada, Las Vegas, NV, USA

**Keywords:** Genitourinary, POCUS, Emphysematous, Epididymo-orchitis, Torsion, Fournier's Gangrene, Point of Care Ultrasound

## Abstract

Point of care ultrasound (POCUS) is a helpful tool for supporting the development of a differential diagnosis for testicular and scrotal pain. It can aid in the diagnosis of critical conditions such as Fournier's Gangrene and testicular torsion. In this case, we describe a 39-year-old man who presented for testicular pain and scrotal swelling who had findings concerning for testicular and epididymal gas on POCUS. This correlated with our computed tomography (CT) findings which were consistent with emphysematous epididymo-orchitis.

## Case presentation

**Figure 1.**
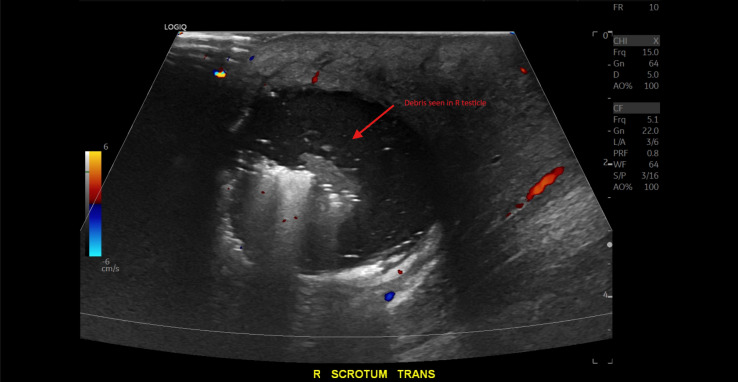
Point of care ultrasound (POCUS) (transverse view) of the right side of the scrotum shows shadowing and a debris pattern consistent with gas in the right testicle. R, right; Trans, transverse.

**Figure 2.**
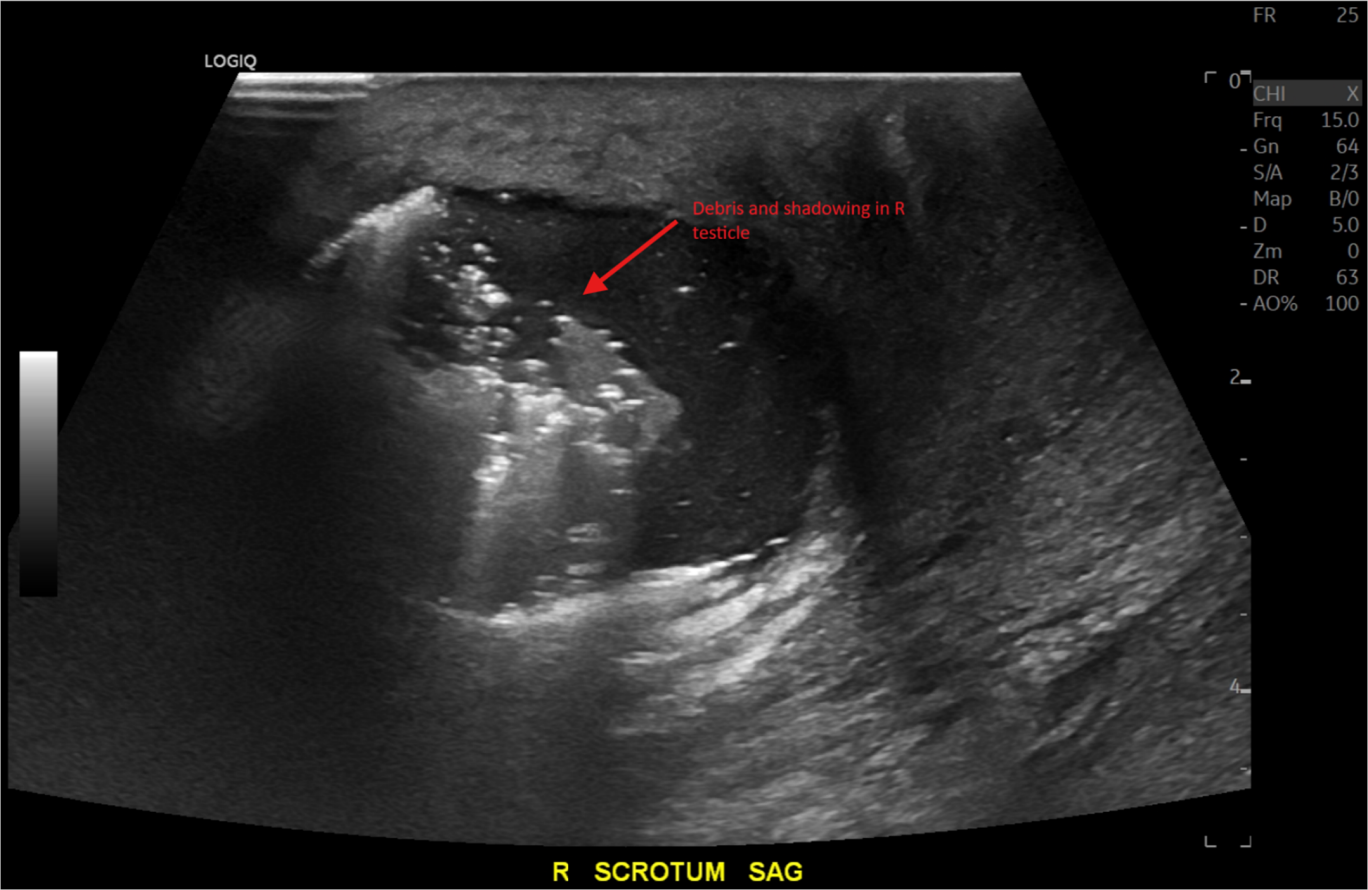
Point of care ultrasound (POCUS) (sagittal view) of the right hemiscrotum demonstrates shadowing and debris. R, right; SAG, sagittal.

**Figure 3.**
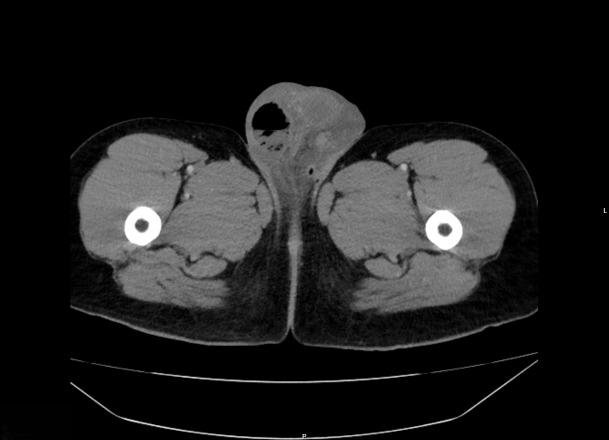
Computed tomography (CT) (transverse plane) of the abdomen and pelvis confirms the presence of gas and debris in the right side of the scrotum, including within the testicle and epididymis.

**Figure 4.**
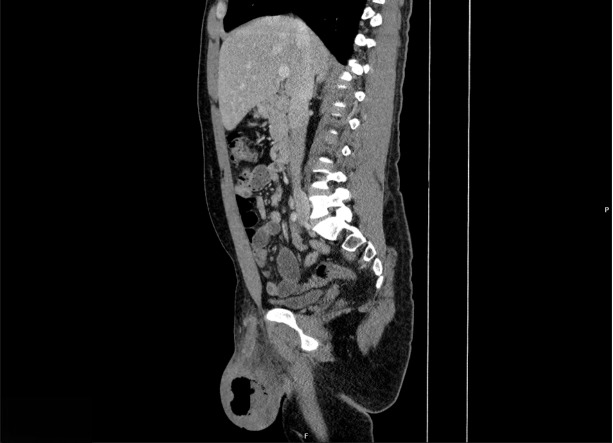
Computed tomography (CT) (sagittal plane) of the abdomen and pelvis confirms the presence of gas and debris in the right side of the scrotum, including within the testicle and epididymis.

A 39-year-old man who was visiting from out-of-state presented to the emergency department with scrotal swelling and pain. The patient reported a “pimple” on the right side of his scrotum one week prior that he attempted to manually squeeze and drain. Afterwards, he began developing significant right-sided testicular and scrotal pain associated with swelling until he was unable to sleep. He had no significant comorbidities—specifically, no history of diabetes or testicular torsion. He had a prior subcutaneous abscess in the same scrotal area in the past for which he received an incision and drainage with improvement in symptoms. In the emergency department, he was afebrile, not tachycardic, and his vital signs were otherwise within normal limits. On examination, his scrotum was significantly swollen with purple skin discoloration severe tenderness to palpation; however, no crepitus was detected and no bullae were observed. Laboratory studies showed CRP of 100.21 mg/L (reference: normal <= 3.00 mg/L), leukocytosis of 21.50 K/mm^3^ (reference range: normal = 3.10-10.20 K/mm3), and mild hematuria with no evidence of urinary tract infection. The Laboratory Risk Indicator for Necrotizing Fasciitis (LRINEC) score was calculated at 5, indicating a mild to moderate risk for possible necrotizing soft tissue infection [1]. POCUS with color Doppler showed normal blood flow to each testicle. It also showed a fluid collection in the right hemiscrotum surrounding the testicle and epididymis. There was hyperechoic debris seen within the fluid collection, as well as dirty shadowing arising from the debris within the fluid collection, suggestive of gas. Importantly, there was no dirty shadowing from the soft tissues of the scrotum itself (Figures 1 and 2). This prompted an emergent CT scan to further characterize the abnormalities seen on POCUS and definitively evaluate for Fournier's Gangrene. The CT scan (Figures 3 and 4) confirmed gas in the testicle and epididymis suggesting a diagnosis of emphysematous epididymo-orchitis. Furthermore, the CT scan did not show gas in soft tissues of the scrotum, thus making necrotizing fasciitis less likely. In consultation with urology, there was a recommended admission for intravenous antibiotics and a possible need for an orchiectomy if the patient failed to improve with medical therapy within the next 24 hours. The patient unfortunately left the department against medical advice to find care closer to home. He was unable to be reached for further comment after leaving; therefore, further progress regarding his case and outcome were unable to be ascertained.

## Discussion

Emphysematous epididymo-orchitis is a rare infection caused by gas-forming bacteria. Its exact etiology is unknown [2]. Some studies suggest that diabetes mellitus is a significant risk factor [3]. Other potential risk factors include concomitant retroperitoneal emphysematous infections adjacent to the origin of the testicular artery or rupture of sigmoid diverticula [3]. Emphysematous epididymo-orchitis is difficult to differentiate from other genitourinary infections as it presents with nonspecific findings such as scrotal swelling, pyuria, bacteriuria, fever, and leukocytosis. POCUS is helpful in evaluating potential causes for the acute scrotum [4,5,6]. We were able to visualize adequate Doppler flow to the testis using POCUS, providing reassurance against testicular torsion in addition to history and physical examination findings. Furthermore, gas arising from the epidermis/fascia of the scrotum and other common sonographic findings for necrotizing fasciitis were not visualized [6]. Crepitus, bullae, and ecchymosis were not seen on physical examination, and the LRINEC score showed only mild-moderate risk for necrotizing infection [1]. Therefore, surgery was not immediately consulted, and we were able to proceed with further diagnostic workup. Gas and debris in and around the epididymis and testicles were apparent on POCUS, which suggested a gas-forming infection of the testicles. This narrowed our differential diagnosis significantly. We were able to expedite our clinical decision making and form an appropriate treatment plan. We ensured antibiotics included covering gas forming organisms, expedited a CT scan, and consulted with urology. This case could have been missed as scrotal cellulitis or abscess leading to an incorrect discharge on oral antibiotics or unnecessary incision and drainage of the skin. Furthermore, the patient could have been rushed to the operating room for an unnecessary surgical intervention for presumed Fournier's Gangrene. Current literature delineates epididymo-orchitis as a separate pathological process from Fournier's Gangrene [7,8]. Unlike Fournier's Gangrene, emphysematous epididymo-orchitis can be treated conservatively with intravenous antibiotics, escalating to surgery if antibiotics fail and a full orchiectomy is necessary. Management should always be guided in close consultation with a urological specialist [7,8].
